# Obesity Development and Signs of Metabolic Abnormalities in Young Göttingen Minipigs Consuming Energy Dense Diets Varying in Carbohydrate Quality

**DOI:** 10.3390/nu13051560

**Published:** 2021-05-06

**Authors:** Mihai Victor Curtasu, Mette Skou Hedemann, Helle Nygaard Lærke, Knud Erik Bach Knudsen

**Affiliations:** Department of Animal Science, Aarhus University, Blichers Alle 20, DK-8830 Tjele, Denmark; mette.hedemann@anis.au.dk (M.S.H.); hellen.laerke@anis.au.dk (H.N.L.); knuderik.bachknudsen@anis.au.dk (K.E.B.K.)

**Keywords:** miniature pigs, dyslipidemia, inflammation, gene expression, carbohydrates, liver metabolism

## Abstract

Consumption of fructose has been associated with a higher risk of developing obesity and metabolic syndrome (MetS). The aim of this study was to examine the long-term effects of fructose compared to starch from high-amylose maize starch (HiMaize) at ad libitum feeding in a juvenile Göttingen Minipig model with 20% of the diet provided as fructose as a high-risk diet (HR, *n* = 15) and 20% as HiMaize as a lower-risk control diet (LR, *n* = 15). The intake of metabolizable energy was on average similar (*p* = 0.11) among diets despite increased levels of the satiety hormone PYY measured in plasma (*p* = 0.0005) of the LR pigs. However, after over 20 weeks of ad libitum feeding, no difference between diets was observed in daily weight gain (*p* = 0.103), and a difference in BW was observed only at the end of the experiment. The ad libitum feeding promoted an obese phenotype over time in both groups with increased plasma levels of glucose (*p* = 0.005), fructosamine (*p* < 0.001), insulin (*p* = 0.03), and HOMA-IR (*p* = 0.02), whereas the clinical markers of dyslipidemia were unaffected. When compared to the LR diet, fructose did not accelerate the progression of MetS associated parameters and largely failed to change markers that indicate a stimulated de novo lipogenesis.

## 1. Introduction

People in developed countries are increasingly adopting unhealthy dietary patterns with poor quality foods based on refined carbohydrates, high levels of fats, and low in dietary fiber (DF). Easy access to such foods, together with a lack of physical activity, has led to an alarming rise in obesity and metabolic syndrome (MetS) [1]. Not only adults suffer from an increased prevalence of obesity, but also childhood obesity that has almost tripled since 1970 [2]. MetS presents complex pathophysiology characterized by abdominal obesity, insulin resistance (IR), hypertension, dyslipidemia, which increases the risk of developing type 2 diabetes (T2D) and cardiovascular disease (CVD) [3].

Obesity and MetS research relied so far primarily on rodent models [4], but recently there has been an increased interest in domestic and miniature swine, a suitable model for disease as demonstrated by the increased use of the Göttingen, Yucatan, and Ossabaw minipig breeds [5,6,7,8]. In addition to similarities regarding genome, anatomy, and digestive physiology, obese pigs are also closely related to obese humans due to their lack of postnatal brown fat, similar metabolic features, cardiovascular system, comparable organ size, and deposition of body fat [5]. Close similarities to human proteins and inflammation responses have also been recently identified [9]. Age and gender of the animal model have an important contribution to the metabolic response in dietary intervention studies [10]. Female minipigs are preferred due to their predisposition to increased weight gain, fat deposition, and dyslipidemic responses compared to males [10,11]. However, animal models studying the onset of obesity in early life are scarce, and the longitudinal aspect of obesity and MetS in juvenile animals has not been well assessed, particularly when subjected to ad libitum feeding.

High-fructose corn syrup used in different pre-packaged foods, fast foods, and soft drinks has been associated with adverse health effects such as obesity development and altered hepatic metabolism through fat accumulation and development of non-alcoholic fatty liver disease (NAFLD) [12,13,14]. Fructose is characterized by a metabolism that may favor hepatic lipogenesis [14]. In contrast to glucose, rapid fructose phosphorylation, and conversion to triose phosphates (glyceraldehyde, dihydroxyacetone phosphate, and glyceraldehyde-3-phosphate), bypasses the key regulatory mechanisms of phosphofructokinase in liver glycolysis [13]. The resulting increase in lipogenic precursors can be mobilized for de novo lipogenesis (DNL), gluconeogenesis, or oxidation pathways [15]. Epidemiological and interventional studies, however, have not been able to clearly support the idea that fructose, compared to other energy-dense nutrients, causes more liver fat accumulation. More likely, the observed steatotic effects of fructose are confounded by obesity and a continuous positive energy balance [16].

The primary objective of the current study was to investigate the physiological effects of high levels of fructose in a diet compared to similar levels of high amylose (HiMaize) starch on obesity development and the risk for developing metabolic abnormalities. We examined the longitudinal effects of the two diets on obesity development and changes in specific MetS biomarkers in a young minipig model over five months of ad libitum intake of a high-risk (HR) fructose based diet compared to a control lower-risk (LR) diet containing the same amount of glucose from HiMaize starch, which contains digestible and fermentable starch [17,18]. It is hypothesized that fructose, because of its metabolic effects, will lead to a more rapid development of obesity and markers of metabolic abnormalities of the young Göttingen Minipigs. This paper is a continuation on the study of Curtasu et al. (2020), where samples collected from this miniature pig model were previously assessed from a metabolomics and gut microbiota profiling perspective [19].

## 2. Materials and Methods

### 2.1. Experimental Diets

Two experimental diets were formulated with 20% of the diet provided as either fructose in the HR diet or as digestible and fermentable starch from HiMaize in the LR control diet (Table S1). The remaining 80% of the diets were similar and provided the fat (35% of energy) and protein (10% of energy), and carbohydrates from starch and fiber. The HR diet formulation resulted in a limited content of dietary fiber (DF) (5% of energy), resembling a typical western-style diet in terms of DF content. The fat source used in this experiment contained the following fatty acid profile: 45.1% saturated, 45.1% monounsaturated, and 9.8% poly-unsaturated fatty acids.

### 2.2. Animals and Experimental Design

Experimental procedures regarding handling of animals were done in accordance with Danish laws and regulations regarding the humane care and use of animals in research (The Danish Ministry of Justice, Act on Animal Experiments no. 474 of 15 May 2014, as stipulated in the executive order no. 12 or 7 January 2016) and according to licenses obtained from the Danish Animal Experimentation Inspectorate, Ministry of Food, Agriculture and Fisheries (Animal experiment permit: 2015−15−0201−00599). Animals were monitored closely on a daily basis by observing the general condition and any manifestations of reduced feeding or drinking desire, lethargy, reduced spontaneous activity, vomiting, fever, diarrhea, labored respiration, or decreased interactions with humans and neighboring pigs. The pigs’ blood sugar levels were monitored bi-weekly using an ear prick glucose test (Accu-Check, Roche Diabetes Care, Inc., Indianapolis, IN, USA). Humane end-points were considered when the condition of the animals could not be remedied by treatment within 1–3 days, the cause could not be clarified or if the pigs were subjected to exceeding strain. None of the animals presented any declining health conditions and all animals completed the trial.

A total of 30 female Göttingen Minipigs (Ellegaard Göttingen Minipigs, Dalmose, Denmark) were delivered at 8 weeks of age in 4 separate blocks over 8 months and were kept isolated from other pigs at the facility for the entire period of the study (Figure S1). In each block, animals were randomly allocated to the diets and pens. Access and handling were permitted only by wearing disposable overalls and latex gloves. For one week, paired housing and restricted feeding of a standard Special Diet Services (SDS, Dietex International, Essex, UK) minipig chow was used according to breeders’ recommendations, followed by gradually transitioning to the experimental diets throughout the coming week. At the start of ad libitum feeding with the experimental diets, animals were separated and housed individually in pens (1.5 × 2.4 m). Wood shavings were provided as bedding, and water provided ad libitum from drinking nipples. The animals had visible access and contact with neighboring animals through open grilled side panels. For the current study, 15 animals per treatment were allotted to the LR and HR diet, respectively. The feed was provided ad libitum for 20 weeks. Feed was weighed out for 2-week periods based on estimated feed intake, and residues collected during this period were pooled and used for calculating daily feed intake. Body weight was recorded every second week for the first six weeks, followed by measurements every fourth week. Every second week, length, chest circumference, and abdominal circumference of the animals were measured. The weight of the animals before the start of dietary transition was not different between the two assigned groups (HR, 3.03 ± 1.9 kg; LR, 3.04 ± 1.9 kg). The animals utilized in this study were previously used for metabolomics and gut microbiota profiling by Curtasu et al. (2020) [19].

### 2.3. Sample Collection

At 4, 12, and 20 weeks of the dietary intervention, animals underwent a broad sample collection procedure. After overnight fasting (16 h) animals were put under anesthesia using 0.1 mL/kg body weight of Zolitil-mixture containing 50 mg/mL tiletamine/zolazepam (Vibrac SA, Carros, France), 2.5 mg/mL butorphanol (Torbugesic^®^ Vet, Scan Vet Animal Health A/S, Fredensborg, Denmark), 12.5 mg/mL ketamine (Ketaminol Vet, Intervet Denmark, Skovlunde, Denmark), and 12.5 mg/mL xylazine (Rompun, Bayer Health Care AG, Leverkusen, Germany). Blood sampling was performed from the jugular vein with the animals in a supine position. A total of 17 mL was collected in vacutainers: 6 mL LiHep, 6 mL K3EDTA, 3 mL LiHepSep, 1 mL K3EDTA/Aprotinin inhibitor (10,000 KIU/mL blood, Nordic Pharma Ltd., Ismaning, Bayern, Germany), 1 mL K3EDTA/DPPIV inhibitor (Vacuette, Greiner Bio-One International, GmbH, Kremsmünster, Austria). Blood plasma was aliquoted for separate analyses and stored at −80 °C after the tubes were centrifuged (12 min at 4 °C, 3300 rpm).

Liver biopsy was performed by moving the animal in a left recumbent position for liver access. The area between the first and fifth teat was shaved and disinfected with 0.5% chlorhexidine solution in 85% alcohol (Abena A/S, Aabenraa, Denmark). Procaine (Procamidor VET, 20 mg/mL, Richter Pharma, AG, Wels, Austria) was injected subcutaneously as a local anesthetic. Ultrasound scanning using a 6–18 MHz linear probe (MyLabTM Five VET, Biosound Esaote Inc., Indianapolis, IN, USA) was performed for guided assistance of the liver biopsy and determining the location of the gallbladder to avoid puncture or damage to surrounding tissues or organs. After a small incision of the skin (5–7 mm), 2–4 biopsies were taken to a total amount of maximum 50 mg of liver tissue with a biopsy pistol (Pro-MagTM I 2.5, Argon Medical Devices Inc., Frisco, TX, USA) and a 14 G × 10 cm needle (Argon Medical Devices Inc., Frisco, TX, USA). Following the procedure, the incision site was closed with surgical staples. Figure S2 presents a visual illustration of the liver biopsy procedure. Afterwards, the right hind leg was cleaned, disinfected, and locally anesthetized, similar to the liver biopsy procedure. After a 15–20 mm skin incision, approximatively 100 mg of subcutaneous adipose tissue (SAT) was collected for gene expression, snap-frozen in liquid N_2_, and stored at −80 °C until analysis. Muscle tissue (50–100 mg) was collected for gene expression from the semitendinosus muscle with the biopsy pistol. A few drops of Streptocillin^®^Vet. (Boehringer Ingelheim Animal Health Nordics A/S, København Ø, Denmark) were administered to the incision site and closed with surgical staples. Both muscle and liver tissues were placed in sterile tubes with RNA later (Sigma-Aldrich Co. LLC, Saint Louis, MO, USA). Fresh feces were collected from the animals immediately after defecation as an effect of the anesthesia. Following these procedures, spot urine samples were collected by placing absorbent tampons on the rear of the minipigs adhesive fabric tape (Omniplast, Hartmann, Baden-Württemberg, Germany).

### 2.4. Analytical Methods

Freeze-dried material in duplicate was used for the chemical analysis of the diets, as previously described [17]. Gross energy (GE) of the diets was determined on a 6300 Automatic Isoperibol Calorimeter System (Parr Instruments, Moline, IL, USA), whereas values of metabolizable energy (ME) intake were calculated based on nutrient intake and the energy conversion factors (FAO) for carbohydrates (17 kJ/g), protein (17 kJ/g), fat (37 kJ/g), and total dietary fiber (8 kJ/g). LiHep plasma was used to measure concentrations of the following metabolites: glucose, fructosamine, lactate, non-esterified fatty acids (NEFA), high-density lipoproteins (HDL), low-density lipoproteins (LDL), total cholesterol (TC), triglycerides (TG), albumin, AST (aspartate transaminase), ALT (alanine transaminase), and GGT (gamma-glutamyltransferase). The analysis was performed using the ADVIA 1650 Chemistry system (Siemens Diagnostics, Tarrytown, NY, USA) according to the manufacturer’s instructions (Siemens Diagnostics Clinical Methods for ADVIA 1650). The same system was used for the analysis of glucose, creatinine, and total protein in urine samples. K3EDTA plasma with aprotinin inhibitor was used for the analysis of metabolic markers: insulin, glucagon, ghrelin (active), glucose-dependent insulin tropic polypeptide (GIP), monocyte chemoattractant protein 1 (MPC-1/CCL2), peptide tyrosine tyrosine (PYY), total glucagon-like peptide-1 (GLP-1), and C-peptide using a Millipore MILLIPLEX MAP Human Metabolic Hormone bead panel kit (HMHEMAG-34K, Merck Millipore, Merck KGaA, Darmstadt, Germany). K3EDTA plasma was used to measure interferon gamma (IFN-γ) and several interleukins (IL-2, IL-4, IL-10, IL-12, IL-18) using the Millipore MILLIPLEX MAP porcine bead panel kit (PCYTMAG-23K, Merck Millipore, Merck KGaA, Darmstadt, Germany). Both kits were run on the Luminex MAGPIX system (Luminex Corporation, TX, USA) according to the manufacturer’s instructions.

### 2.5. Gene Expression Analysis of Liver, Muscle, and Subcutaneous Adipose Tissue (SAT) by Real-Time Reverse Transcriptase-Polymerase Chain Reaction (RT-PCR)

Liver, muscle, and SAT were analyzed for the expression of 14, 11, and 12, respectively, selected gene transcripts by using gene-specific probes and porcine-specific primers (Table S2). Total RNA extraction from liver tissue was performed using the NucleoSpin RNA Plus kit (Macherey-Nagel GmbH & Co., KG., Duren, Germany) according to the manufacturer’s instructions. Muscle and SAT total RNA was extracted using TRI Reagent^®^ Solution (Ambion, Applied Biosystems, Stockholm, Sweden) following the manufacturer’s protocol. RNA transcription, cDNA synthesis, and RT-PCR quantification were done as described in Supplementary Materials. Glyceraldehyde 3-phosphate dehydrogenase (GAPDH), β-actin, and hypoxanthine phosphoribosyltransferase 1 (HPRT1) were tested as housekeeping genes (HKG). Gene expression data was obtained as Ct values and used to calculate ΔCt values as the difference between Ct of the target gene and mean Ct of HKG (Ct value represents the cycle number at which logarithmic plots cross a calculated threshold). Liver GAPDH exhibited changes concerning the two diets and also with time development. As a result, β-actin and HPRT1 were used as mean HKG for liver tissue, whereas β-actin and GAPDH were used as mean HKG for muscle and SAT. Relative gene expression was determined using the (1 + efficiencies)-ΔΔCt method, were ΔΔCt = ΔCttarget-ΔCtLRweek4. Expression in muscle and SAT expression of C-reactive protein (CRP) and muscle leptin receptor (LEPR) expression were measured close to the detection limit, and as a result, values are not reported. Results were reported as fold changes.

### 2.6. Calculations and Statistical Analyses

Porcine obesity index (POI) was calculated [7]:

POI = (π × (1/3) × BS × (Abr2 + Cr2 + Ab × Cc))/BS(1)

where BS represents body size (length), Abr abdomen radius, Cr chest radius, Ab abdomen circumference, and Cc chest circumference.

Body surface area (BSA) was calculated using the proposed formula for miniature swine [20]:

BSA = 0.121BW^0.575^ (2)

where BW represents body weight. Calculations of the homeostatic model assessment for insulin resistance (HOMA-IR) and beta-cell function (HOMA-B) were done as previously described [21].

Statistical analysis of the weight development, feed intake, biochemical parameters, blood biomarkers, anthropometric measurements, and gene expression were performed using Statistical Analysis Software (SAS, version 9.4, SAS Institute Inc., Cary, NC, USA). Diet, time, and their interaction effects were analyzed using a Linear Mixed Model for repeated measurements:

(3) $$Y_{ijkl}=\mu +\alpha_{i}+\beta_{j}+\alpha \beta_{ij}+\gamma_{k}+\gamma_{l}+\varepsilon_{ijkl}$$

where *Y_ijkl_* is the analyzed variable; *µ* is the overall mean; *α*_i_ represents the effect of diet (*i* = LR, HR); *β_j_* is the collection time (*j* = 4, 12, 20); *αβ_ij_* is the interaction between diet and time; *γ_k_* is the random effect of the block (*k* = 1, 2, 3, 4), and *γ_l_* is the random component of the individual animal (*l* = 1, 2,…, 30). Data was modeled to account for the repeated measurements of time using an autoregressive covariance structure of order 1. The *ε_ijkl_* component represents the residual error. Tissue gene expression data was analyzed using a similar model where the collection time only reflects two time points (*j* = 4, 20).

Data are presented as Least Square Means (LSM) ± standard error of mean (SEM). Significance level is assumed for *p* < 0.05, whereas 0.05 ≤ *p* ˂ 0.10 is describing tendencies. Spearman correlations of body weight and plasma albumin were performed in RStudio Version 1.1.456 (RStudio Inc, Boston, MA, USA) using the cor.test function. Due to other experimental analysis performed on the adipose tissue, only 8 animals were included in the gene expression analysis at week 4 compared to 15 animals at week 20. The hypothesized changes in biomarkers of dyslipidemia were based on previous analysis done on ad libitum vs. restrictive fed pigs in a MetS context [22]. Sufficient statistical power (*α* < 0.05; *β* = 0.80) was expected from 6–8 pigs completing the study according to the power calculations for triglycerides and total cholesterol.

## 3. Results

### 3.1. Diet Composition

The experimental diets were formulated to provide on a dry matter (DM) basis equal amounts of energy from fat (174–177 g/kg DM) and protein (113–119 g/kg DM), but to differ regarding content and sources of available carbohydrates and DF (Table 1). Available carbohydrates in the HR diet added up to 555 g/kg DM, out of which 225 g/kg DM was fructose. In the LR diet, the available carbohydrates were lower, 424 g/kg DM mostly as starch (415 g/kg DM), but higher in total DF (188 g/kg DM); the difference was in RS (89 vs. 2 g/kg DM), whereas the non-starch polysaccharides (NSP) content was practically similar in the two diets (73 and 69 g/kg DM). On a pure carbohydrate basis and expressed as monosaccharides, the difference between the HR and LR diet was that the HR diet provided 225 g/kg DM as fructose that substituted 222 g/kg DM glucose monosaccharides from starch in the LR diet. Overall, gross energy (20.3–20.7 MJ/kg DM) was comparable between the two diets.

### 3.2. Nutrient and Energy Intake

**Figure 1:**
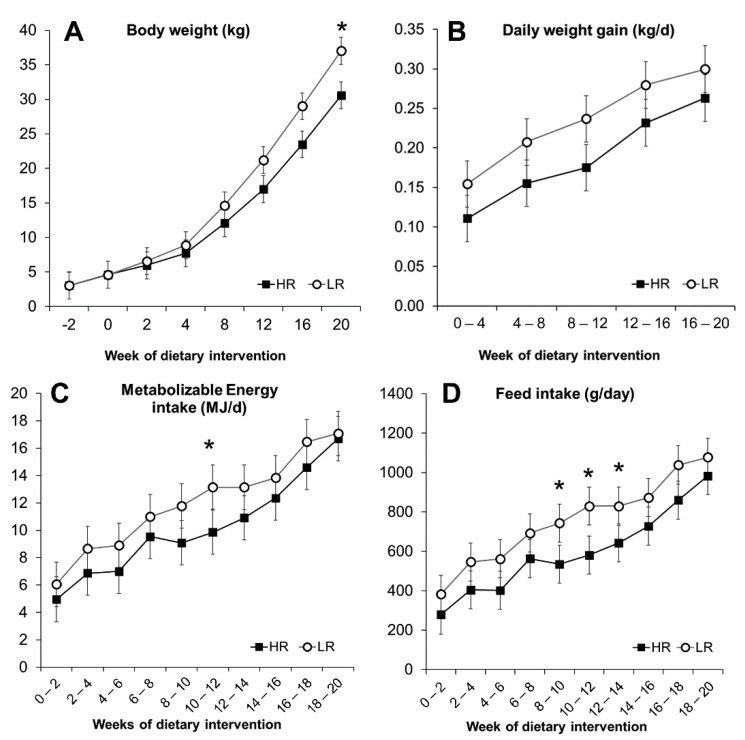
Development parameters of Göttingen Minipigs fed ad libitum a high-risk (HR, *n* = 15) and a lower-risk (LR, *n* = 15) diet. Body weight (**A**), daily weight gain (**B**), calculated metabolizable energy intake (**C**), and daily feed intake (**D**). Results are expressed as LS means, error bars indicating SEM. Significant difference (* *p* < 0.05).

Over the 20-week intervention trial, the intake of metabolizable energy (ME) calculated based on feed intake, was on average similar (*p* = 0.11) as the HR group had an intake of 10.2 MJ/d, compared to the 12 MJ/d of the LR group and only a notable difference was observed at weeks 10–12, as seen in Figure 1C. The animals on the two diets consumed, on average, the same amount of available carbohydrates, whereas for all other nutrients, the intake was higher in the LR group primarily because of higher feed intake (Table 2). Proportionally, animals on the HR diet ingested 9% more energy from carbohydrates, but less from fat, protein, and DF (3.2%, 1.4%, and 4.4%, respectively) compared to the LR group.

### 3.3. Obesity Development and Morphometric Measurements

**Figure 2:**
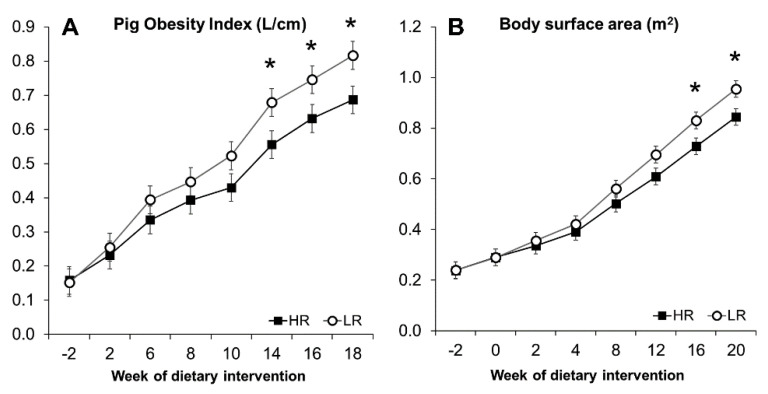
Pig obesity index (**A**) and body surface area (**B**) calculated from morphometric measurements. Results are expressed as LS means, error bars indicating SEM. Significant difference (* *p* < 0.05).

The minipigs gained weight at a comparable rate during the experiment, regardless of the dietary intervention. Body weight (BW) and daily weight gain (DWG) increased significantly over 20 weeks of dietary intervention (*p* < 0.001), as seen in Figure 1A,B. During the first two weeks, HR and LR groups had a feed intake (FI) of 287 and 382 g/day, respectively, increasing to 984 g for the HR group and 1077 g for the LR during the last two weeks (Figure 1D). A primary effect of the diet was observed with the LR group, where overall measured FI was higher (*p* = 0.023; Figure 1D). The higher FI, however, did not translate into differences in overall DWG (*p* = 0.103; Figure 1B), BW (*p* = 0.33; Figure 1A), or the calculated POI (*p* = 0.14) and BSA (*p* = 0.25) measurements (Figure 2). The calculated POI and BSA measurements showed an increased adiposity for minipigs consuming the LR diet only during the last period of the intervention (Figure 2) as well as a difference in BW at week 20 (Figure 1A). Measurements of length, chest circumference, and abdominal circumference are shown in Figure S3.

### 3.4. Plasma and Urine Biomarkers of MetS and Biomarkers of Inflammation

**Figure 3:**
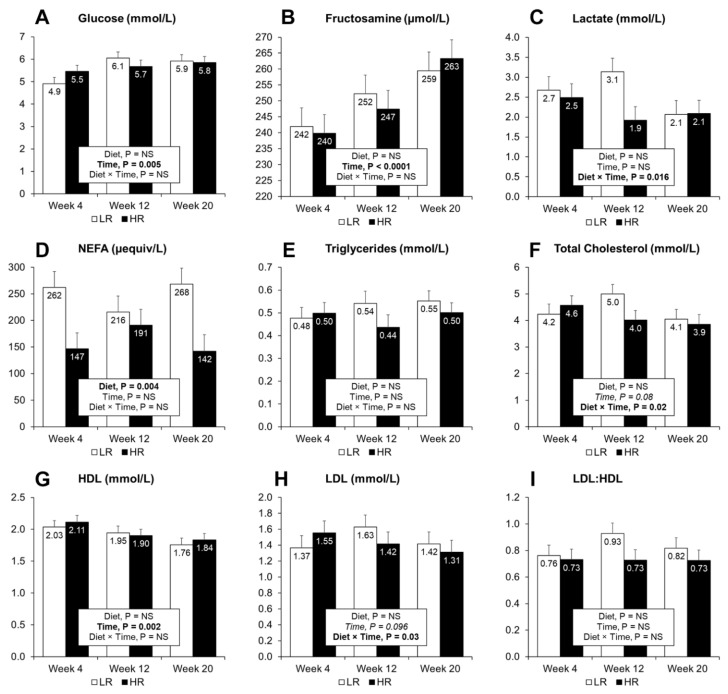
Plasma concentrations of biomarkers after overnight fasting. Measurements of glucose (**A**), fructosamine (**B**), lactate (**C**), NEFA (**D**), triglycerides (**E**), total cholesterol (**F**), high-density lipoprotein cholesterol (**G**), low-density lipoprotein cholesterol (**H**) and the LDL to HDL ratio (**I**), were taken at 4, 12 and 20 weeks during the dietary intervention. Results are expressed as LS means, error bars indicating SEM. NS, indicates non-significance at *p* > 0.05. Significant difference at *p* < 0.05.

Plasma and urine metabolites measured after overnight fasting are presented in Figure 3 and Table 3, respectively. Plasma glucose (*p* = 0.005) and fructosamine (*p* < 0.001) increased, whereas the level of HDL cholesterol decreased with time (*p* = 0.002). Diet did not affect other measured plasma metabolites except for NEFA (*p* = 0.003), where higher levels were observed in the LR group. A tendency for decreasing cholesterol level was observed with time (*p* = 0.08), and neither diet nor time affected triglyceride levels. The HR diet led to increased urinary creatinine (*p* < 0.001) and glucose (*p* = 0.005) output, but did not affect total protein content (Table 3). However, when expressed as protein to creatinine ratio (PCR), lower values were observed at weeks 12 and 20 with the HR group.

**Figure 4:**
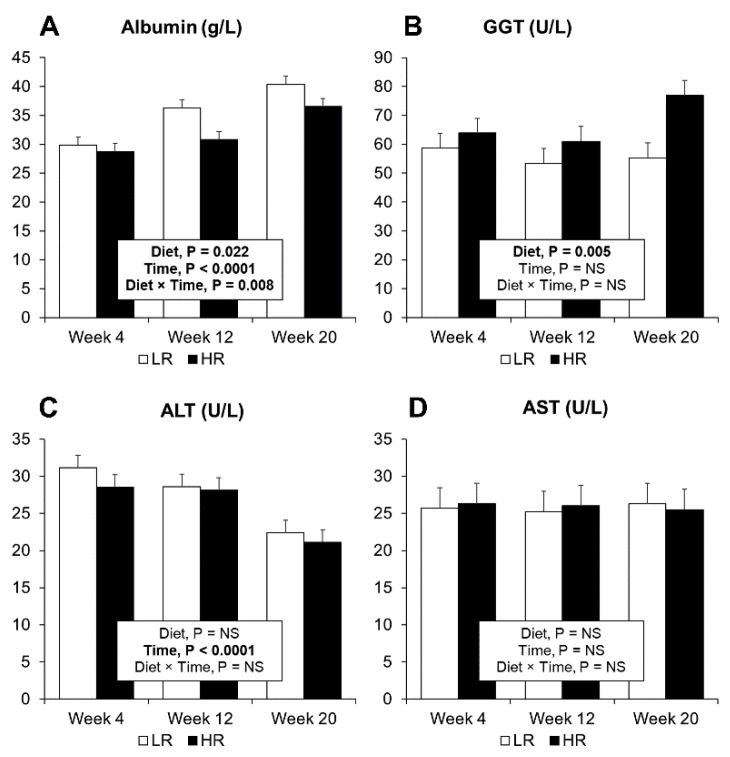
Clinical plasma biomarkers for liver function. (**A**) Albumin measurements. (**B**) GGT (gamma-glutamyl transferase). (**C**) ALT (alanine transaminase). (**D**) AST (aspartate transaminase). Results are expressed as LS means, error bars indicating SEM.

Plasma albumin was observed to increase in both the LR and HR diets during the dietary intervention (Figure 4A). Furthermore, the LR diet increased albumin levels when compared to the HR diet (Figure 4A). In both dietary groups, strong correlations between BW and measured albumin levels were observed (Figure S4). Two liver transaminases: ALT, AST (Figure 4C,D), together with GGT were assessed in plasma, where only GGT had a diet effect with higher levels observed in the HR diet compared to the LR (Figure 4B). ALT levels decreased with time in both dietary groups (Figure 4C).

Fasting levels of circulating plasma hormones are presented in Table 4. Insulin (*p* = 0.03), total GLP-1 (*p* = 0.04), and glucagon (*p* < 0.0001) increased during the dietary intervention; GIP levels decreased over time (*p* < 0.0001), whereas C-peptide, PYY, and ghrelin levels were unaffected. Higher levels of glucagon (*p* = 0.01) were observed with the HR diet, whereas lower values of PYY were present when compared to the LR diet (*p* = 0.0005). HOMA-IR indicated a time-dependent increase (*p* = 0.02) with no difference between the two diets. None of the plasma inflammatory markers (IFGg, IL2, IL4, IL10, IL12, IL18) measured in this experiment exhibited a response to the diets (Table S3), and only IL12 decreased from week 4 to week 20 (*p* = 0.0003).

### 3.5. Tissue Gene Expression

**Figure 5:**
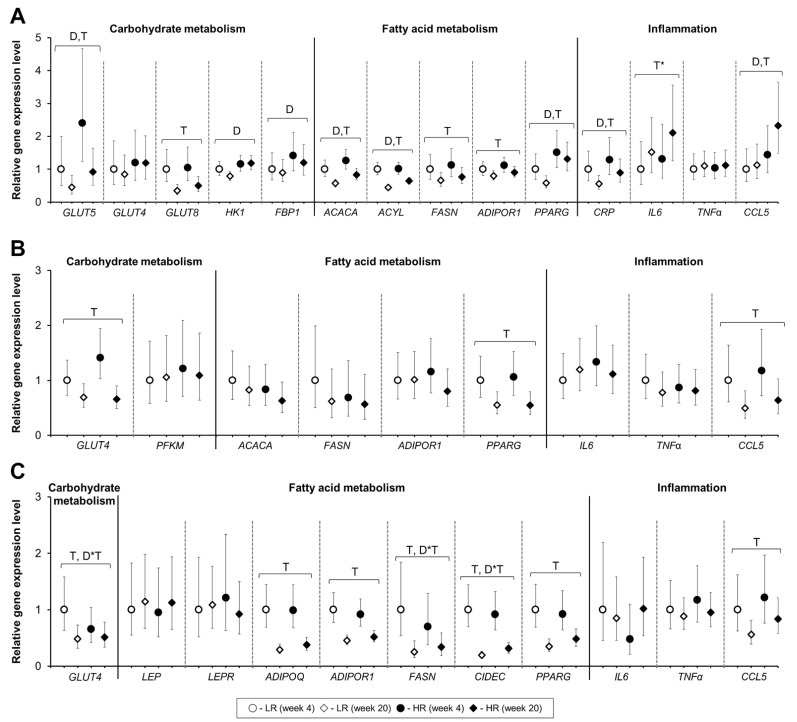
Gene expression of selected genes in the liver (**A**), muscle (**B**), and subcutaneous adipose tissue (**C**) collected at week 4 and week 20 of the dietary intervention from ad libitum fed female Gottingen Minipigs. Solute Carrier Family 2 (Facilitated Glucose/Fructose Transporter) Member 5 (GLUT5); Solute Carrier Family 2 (Facilitated Glucose Transporter) Member 4 (GLUT4); Solute Carrier Family 2 (Facilitated Glucose Transporter) Member 8 (GLUT8); Hexokinase 1 (HK1); Fructose-Bisphosphatase 1 (FBP1); Phosphofructokinase, Muscle (PFKM); Acetyl-Coenzyme A Carboxylase Alpha (ACACA); ATP-citrate lyase (ACYL); Fatty Acid Synthase (FASN); C-C Motif Chemokine Ligand 5/RANTES (CCL5); Adiponectin (ADIPOQ); Adiponectin receptor 1 (ADIPOR1); Leptin (LEP); Leptin Receptor (LEPR); Cell Death-Inducing DFFA Like Effector C (CIDEC); Peroxisome proliferator-activated receptor gamma (PPARG); C-reactive protein (CRP); Interleukin 6 (IL6); Tumor Necrosis Factor (TNFα). Results are presented as ddCT values with 95% CI, reported relative to the LR diet at week 4. LR, lower-risk diet. HR, high-risk diet. Number of animals-liver: LR week 4 (*n* = 10), LR week 20 (*n* = 15), HR week 4 (*n* = 11), HR week 20 (*n* = 15). Number of animals-muscle: LR-HR week 4 (*n* = 14), LR-HR week 20 (*n* = 15). Number of animals-subcutaneous adipose tissue: LR-HR week 4 (*n* = 8), LR-HR week 20 (*n* = 15). D = effect of diet (*p* < 0.05), T = effect of time (*p* < 0.05). D*T = effect of interaction diet and time (*p* < 0.05). T* = tendency effect of time (0.05 ≤ *p* ˂ 0.10).

The relative hepatic gene expression of Solute Carrier Family 2 Member 5 (*GLUT5*), hexokinase 1 (*HK1*), fructose-biphosphatase 1 (*FBP1*), acetyl-Coenzyme A carboxylase alpha (*ACACA*), ATP-citrate lyase (*ACYL*), C-reactive protein (*CRP*), and peroxisome proliferator-activated receptor gamma (*PPARG*) increased with the HR diet (Figure 5A). The hepatic gene expression of C-C Motif Chemokine Ligand 5/RANTES (*CCL5*) was increased with the HR diet at the end of the study (Figure 5A). Diet had no significant effect on the relative gene expression in muscle or SAT (Figure 5B, 5C). A significant diet and time interaction was observed in the SAT for Solute Carrier Family 2 Member 4 (*GLUT4)*, fatty acid synthase (*FASN),* and Cell Death-Inducing DFFA Like Effector C (*CIDEC*) where higher expression levels were observed in response to the LR diet at week 4 of the dietary intervention. Time had a more pronounced effect than diet on gene expression in all three tissues analyzed. Irrespective of the diet, the relative expression of *GLUT5*, Solute Carrier Family 2 Member 8 (*GLUT8*), *ACACA*, *ACYL*, *FASN*, *ADIPOR1*, and *CRP* decreased from week 4 towards week 20 in the liver. Only *CCL5* showed a higher expression levels at the end of the experiment (*p* = 0.02), while *IL6* show a tendency for higher expression at week 20 (*p* = 0.06) and no effect of the diet was observed. Three genes had lower expression in the muscle at the end of the dietary intervention: *SLC2A4*, *CCL5*, and *PPARG* (*p* < 0.01). Genes expressed in the SAT followed similar tendencies with a decrease in expression seen for *GLUT4*, *ADIPOR1*, adiponectin (*ADIPOQ*), *FASN*, *CIDEC*, *PPARG*, and *CCL5* after 20 weeks of dietary intervention.

## 4. Discussion

In the context of carbohydrate quality and quantity consumption, the rationale behind the two experimental diets was that the LR diet should resemble a typical human diet recommended in Western societies, high in complex carbohydrates such as starch and dietary fiber, whereas the HR diet, in contrast, should resemble a Western-style diet with the monomeric sugar substituting complex carbohydrates. The two diets also contributed with equal amounts of energy from fat and protein; the level of protein was reduced to redirect energy from lean tissue accretion to adipose tissue storage and to diminish the muscle mass for glucose regulation [23]. Thus, the intention was to study the higher expected risk presented by the fructose ingredient substituting HiMaize starch that contains a mix of digestible and fermentable starch [18]. It is expected that the fermentation of RS to short-chain fatty acids (SCFA) will influence satiety hormones (GLP-1 and PYY) [24,25] and lower the risk of developing obesity and metabolic signs of MetS [26,27,28].

In the current study, we used ad libitum intake as has been employed successfully in several studies on swine [22,29,30] to mimic the behavior of humans with tendencies towards overeating [31]. We were expecting that the higher fermentation of RS in the LR diet would have a direct effect on satiety control mechanisms [25] and thereby influence the ad libitum feed intake. Previously published data on gut microbiota revealed a higher abundance of microbiota associated with acetate production (*Bacteroidetes* and *Ruminococcus*) and fecal and plasma SCFA in the LR diet [19]. This, together with the higher release of PYY throughout the trial, is in agreement with previous studies in swine [24], but in contrast to our expectations where the feed intake of the LR group was higher than of the HR group. A reason for that could be the higher acetate production in the LR group [19] causing acetate-mediated hyperphagia in an ad libitum context, as recently found in a rodent study [32]. Other studies applying ad libitum feeding with RS to pigs have also failed to influence voluntary feed intake and carcass quality compared to a low-RS diet [33,34]. Moreover, although adiposity in rodents, nonhuman primates, and humans has been strongly correlated with plasma leptin concentrations [35], our data on mRNA expression levels of leptin and leptin receptor in the subcutaneous adipose tissue did not show any significant difference between the two diets. Therefore, we believe that other mechanisms than satiety hormones may be responsible for the higher feed intake of LR diet compared to HR diet, such as differences in palatability between fructose and HiMaize.

Although fructose has a potential DNL effect, results from this and other recent studies provide conflicting evidence. At low doses, the small intestine is the primary organ for dietary fructose clearance, whereas, at high doses, the clearance capacity is saturated, resulting in spillage to the colonic microbiota and the liver [36]. Gene expression analysis confirms that *GLUT5* is not only expressed and facilitates fructose absorption at the intestinal level, but also in the hepatic tissue where higher expression levels of *GLUT5* were measured with the HR diet. Other transporters analyzed, such as *GLUT4* or *GLUT8,* did not respond to the intake of fructose, confirming that *GLUT5* is a significant transporter of fructose in the liver [37]. Fatty acid synthesis is favored through the regulatory effects of SREBP-1c and ChREBP on fatty acid synthase (*FASN*) and acetyl-CoA carboxylase (*ACACA*) [16,38]. Hepatic gene expression of ATP-citrate lyase (*ACLY*) and *ACACA* were both increased by the presence of dietary fructose after 20 weeks of dietary intervention. However, the expression of *FASN* was not increased by the HR diet in the hepatic, muscular, or adipose tissue and a rise in lactate, a by-product of DNL from fructose, in the systemic circulation [13] was not observed in this study. Interestingly, the HR diet lowered albumin secretion from the liver, which could indicate impaired liver function, as seen previously in rats fed high-fat-high-fructose and ethanol diets [39]. Glucagon levels were higher throughout the trial in the HR diet compared to the LR diet. However, differences between the groups are not sufficient to indicate a release due to a state of hypoglycemia as the levels of glucose and fructosamine (indicating long-term glucose levels) were unaffected. A potential explanation might come from the different carbohydrate sources, as the LR diet provides directly digestible glucose from starch, whereas the metabolism requires extra steps for the hepatic release of glucose synthesized from fructose. Similar increases of glucagon levels have been reported in juvenile minipigs exposed to high-fat-high-fructose/sucrose diets without visible changes in glucose, fructosamine, or insulin levels [40].

Irrespective of the carbohydrate quality of the diets, there was a down-regulation of *GLUT4* expression in muscle and SAT, towards the end of the dietary intervention, which indicates an early progression toward insulin resistance and T2D pathogenesis as has been found in adipose tissue *GLUT4* knockdown mice that developed insulin resistance [41], whereas overexpression of *GLUT4* in adipocytes reduced fasting hyperglycemia and prevented insulin resistance [42]. Branched-chain amino acids (BCAA, leucine, valine) are linked to insulin resistance via *GLUT4* [43] and the accumulation of BCAA and BCAA degradation products found in our metabolomic study [19] could be an early sign of insulin resistance as further indicated by the significantly increased plasma levels of glucose (*p* = 0.005), fructosamine (*p* < 0.001), insulin (*p* = 0.03), and HOMA-IR (*p* = 0.02) in both groups from week 4 to week 20. In spite of these changes in metabolic biomarkers, the minipigs developed only some of the hallmarks of MetS; increased body weight, BSA, and POI with visible deposition of subcutaneous fat, alterations in fasting glucose, and insulin responses, and decreasing levels of HDL cholesterol. Total cholesterol, triglycerides, and LDL, however, showed no dysregulation, in contrast to other studies where minipigs developed more severe signs of diet-induced MetS in high-fat diet trials with or without cholesterol supplementation [8,44,45,46]. A confounding effect of increasing intake of dietary fat, which deregulates the synthesis of fatty acids [44] cannot be excluded as several hepatic and adipose tissue genes related to fatty acid metabolism (*ACACA*, *ACYL*, *FASN*, *CIDEC*, and *PPARG*) expressed were downregulated with time. In this context, the age of animals could play a role in the rate of disease development. Post-weaned and growing swine have a higher capacity for fat synthesis as lipogenic enzymes reach a plateau during aging [47]. Furthermore, swine generally have an increased innate capacity for adipose tissue expansion independent of adipocyte count, whereas more mature animals, like the case in humans [48], are less flexible and therefore more prone to be affected by an energy-dense dietary intervention, as seen in swine models of sarcopenic obesity [49] and MetS [8]. A contributing factor for dyslipidemia development is the level and type of fat. Although the dietary fat levels used in this study were high (17.4–17.7%) compared to a conventional pig diet, and it was insufficient for the development of dietary dyslipidemia. Other experiments with pigs have used over 30% fat [8,50] or have accelerated dyslipidemia with cholesterol and sodium cholate [50]. The gene expression of adiponectin in the subcutaneous adipose tissue revealed a time-driven decline as well as *PPARG*, which is an essential mediator of adiponectin expression in the adipose tissue [51]. This, together with the increasing levels of insulin and glucose, could be interpreted as the minipigs evolving towards a state of chronic obesity with signs of insulin resistance. These results are in agreement with current knowledge linking obesity to the development of T2D and CVD [52,53].

Contrary to our expectations, the circulating levels of plasma inflammation markers did not reveal any signs of inflammation during the 20 weeks of dietary intervention irrespective the dietary treatments. A similar situation was observed in juvenile Ossabaw pigs, where a high-fat diet induced dyslipidemia, IR, and hypertension, but did not increase visceral or subcutaneous adipose tissue inflammation or circulatory levels of cytokines [54]. In the liver tissue, however, the exposure to both diets over 20 weeks of dietary intervention increased the expression of *CCL5* (RANTES) and *IL6*, but decreased the expression of *CRP*. However, the presence of fructose seems to influence hepatic gene expression differently with the upregulated expression of *CRP* and *CCL5* by the HR diet compared to the LR diet. *CCL5* cytokine plays an important role in recruiting leukocytes at specific inflammatory sites and has been linked to the progression of hepatic inflammation and fibrosis in the context of NAFLD/NASH [55]. *CRP* is predominately produced by the liver as a response to inflammation and tissue damage and the presence of dietary fructose seems to increase circulatory levels of CPR, as also seen in a previous Göttingen minipig study [56]. The two well-known clinical indicators for hepatitis, ALT, and AST, however, showed no effect of diet as also report by Schumacher-Petersen et al. in male Göttingen pigs [57], and in other fructose interventions in minipigs [58,59]. On the other hand, GGT was increased with the HR diet, as was also reported with a fructose diet in a NASH minipig model, although not causing significant steatosis, but only foamy macrophage-like cells [40]. Taken together, the long-term exposure to fructose showed no increase in expression levels of the specific DNL related genes nor an increase in circulatory lactate levels. Although fructose increased CCL5 and CRP expression level in the liver as well as circulating GGT levels, it would be difficult to confirm the presence of an inflammatory state at the hepatic level without more extensive analyses or liver histology. In another study, severely obese Gottingen Minipigs have been described to avoid the development of hepatic steatosis through fructose due to their higher capacity of adipose tissue expansion and protection by developing a metabolically healthy obese phenotype [60]. Finally, dyslipidemia is key to the development of liver disease [54,55], and according to more recent studies in minipigs, fructose does not significantly influence hepatic fatty acid synthesis when compared to sucrose [40].

### Study Limitations and Strengths

The present study has several strengths, but also potential weaknesses that should be considered. Although ad libitum feeding can be advantageous for the acceleration of obesity, the continuous access to energy might disrupt differences between the pre- and post-absorptive phase, thereby putting less stress on the regulatory mechanisms of the metabolism and disease development compared to regular meal feeding. Ad libitum feeding also disrupted our capacity to conduct a successful meal glucose or insulin tolerance test, as the constant access to feed de-regulated the capacity of the animals to intake large portions of feed. Tissue biopsies were collected in a fasting state, and while it is well established that a fasting state can deregulate gene expression in the liver, interpretation of the results should be done considering that these animals were for the full duration of the experiment subjected to ad libitum feeding. Furthermore, differences between human and swine liver metabolism should be considered regarding TG secretion and inflammation. The swine liver plays a minor role in DNL compared to the adipose tissue [61,62], and this might explain why fructose did not stimulate the *FASN* expression and organ fat deposition. Animal model studies commonly utilize a small number of animals per group due to increased costs of the animal model and maintenance. One strength of this study was the large number of replicates utilized. Moreover, the collection of repeated organ biopsies on the same animal is not common practice with miniature swine, and it provided new information on tissue metabolism in young and developing Göttingen Minipigs. However, the results should be interpreted with care in the absence of information on enzymatic activities occurring in the liver, muscle, or adipose tissue and the absence of histological data on these tissues.

## 5. Conclusions

We observed that ad libitum intake of high-energy diets with fructose or HiMaize both favored a rapid fat accumulation inducing an obese phenotype with increased fasting glucose, signs of insulin deregulation in this juvenile minipig model. Furthermore, the high-level fructose intake failed to induce a higher state of dyslipidemia or other markers of metabolic syndrome when compared to the HiMaize based diet. Given the young age of these minipigs, relative metabolic flexibility appears to be still present when feeding energy-dense diets for 20 weeks, and the disease phenotype is not clearly established regarding MetS.

## Figures and Tables

**Table 1:** Chemical composition of the experimental diets.

	LR	HR
Chemical composition (g/kg DM ^1^)		
DM (g/kg, as-fed basis)	917	913
Ash	63	62
Protein (N × 6.25)	119	113
Fat	177	174
Available carbohydrates	424	555
Sugars	9	233
Fructose	0.6	225
Glucose	1.2	0.8
Sucrose	7	7
Starch	415	322
Total dietary fiber ^2^	188	100
Total NSP (soluble NSP) ^3^	73 (15)	69 (8)
RS ^4^	89	2
AXOS ^5^	3	5
Fructans	5	6
Klason lignin	18	18
Gross energy (MJ/kg DM)	20.3	20.7

^1^ Dry matter; ^2^ Total NSP + fructans + RS + lignin + AXOS; ^3^ Non-starch polysaccharides; ^4^ Resistant starch; ^5^ Arabinoxylan-oligosaccharides.

**Table 2:** Average nutrient intake over a 20-week intervention period and relative energy contribution of diet components.

	LR ^1^	HR ^1^	SEM	*p*-Value
Nutrient intake (g/day) ^2^				
Dry matter	694	548	78	0.023
Available carbohydrates	295	304	39	0.775
Protein	83	62	9	0.006
Fat	123	95	14	0.015
Total dietary fiber ^3^	130	55	13	<0.0001
Relative energy contribution (%) ^4^				
Carbohydrates	41.8	50.8		
Fat	37.9	34.6		
Protein	11.7	10.3		
Total dietary fiber	8.7	4.3		

^1^ Data presented as LS means; LR (*n* = 15), HR (*n* = 15). ^2^ Calculated from feed intake. ^3^ Total NSP + fructans + RS + lignin + arabinoxylan-oligosaccharides. ^4^ Calculated from nutrient intake using the FAO energy conversion factors for carbohydrates (17 kJ/g), protein (17 kJ/g), fat (37 kJ/g), and total dietary fiber (8 kJ/g).

**Table 3:** Urinary concentrations of metabolites after overnight fasting.

				*p*-Value ^2^		
	Week	LR ^1^	HR ^1^	Diet	Time	Diet × Time
Creatinine (µmol/L)	4	4.7 (3.6, 6.1)	10 (7.4, 13.6)	0.0002	NS	0.02
	12	6.8 (5.3, 8.9)	9.4 (7, 12.6)			
	20	5.9 (3.9, 6.8)	12 (9.4, 15.3)			
Glucose (mmol/L)	4	0.4 (0.1, 1.3)	1.8 (0.4, 7.2)	0.004	0.03	NS
	12	0.4 (0.1, 1.4)	4.7 (1.2, 17.8)			
	20	3.8 (1.2, 12.1)	3.6 (1.3, 10.4)			
Total Protein (mg/L)	4	74.9 (46, 123)	136.1 (76, 243)	NS	NS	0.03
	12	169.9 (104, 278)	75.6 (43, 134)			
	20	150.5 (92, 246)	150 (97, 233)			
Glucose: Creatinine Ratio ^3^	4	0.1 (0.03, 0.3)	0.2 (0.05, 0.7)	NS	NS	NS
	12	0.1 (0.02, 0.2)	0.2 (0.06, 0.7)			
	20	0.4 (0.11, 1.1)	0.3 (0.11, 0.8)			
Protein Creatinine Ratio	4	17.1 (11.3, 22.9)	17.2 (10.8, 23.6)	0.004	NS	0.05
	12	27 (18.2, 35.9)	4.5 (−5.7, 14.8)			
	20	27.7 (18.6, 36.8)	13.2 (4.6, 21.9)			

^1^ Data presented as LS means; LR (*n* = 15), HR (*n* = 15); 95% CI are given for analysis with logarithmically transformed data; ^2^ non-significant (NS), *p* > 0.1.

**Table 4:** Plasma concentrations of circulating hormones.

					*p*-Value ^4^		
	Week	LR ^1^	HR ^1^	SEM	Diet	Time	Diet × Time
C-Peptide **^2^**	4	28	27.2				
	12	34.1	27.5	3.6	NS	NS	NS
	20	30	27.9				
Ghrelin **^2^**	4	11.3	15				
	12	13.6	10.6	3.2	NS	NS	NS
	20	11.1	16.5				
GIP **^2^**	4	92.3	94.2				
	12	70.8	55.7	8.4	NS	<0.0001	NS
	20	43.8	50.3				
GLP1T **^3^**	4	207.6	234.6				
	12	258.8	255.2	18.8	NS	0.04	NS
	20	236	236.1				
Glucagon **^2^**	4	163.1	202.5				
	12	228.7	302.1	25.5	0.01	<0.0001	NS
	20	213.8	295.5				
PYY **^2^**	4	281.8	234				
	12	368.7	227.3	35.7	0.0005	NS	NS
	20	299.3	170.1				
Insulin (pmol/L)	4	28.0	31.2				
	12	33.9	36.4	4.6	NS	0.03	NS
	20	38.9	43.5				
HOMA-IR	4	0.52	0.59				
	12	0.66	0.71	0.09	NS	0.02	NS
	20	0.76	0.84				
HOMA-B	4	63.6	60.4				
	12	52.3	60.3	8.7	NS	NS	NS
	20	56.2	67.8				

^1^ Data presented as LS means; LR (*n* = 15), HR (*n* = 15); ^2^ measured in pg/mL; ^3^ total GLP-1; ^4^ non-significant (NS), *p* > 0.1.

## Data Availability

The data presented in this study are available on request from the corresponding authors.

## Supplementary Materials

The following are available online at https://www.mdpi.com/article/10.3390/nu13051560/s1, Table S1. Feed ingredients; Table S2. RT-PCR gene expression assays; Table S3. Plasma concentrations (ng/mL) of inflammation biomarkers after overnight fasting; Figure S1. Flow chart presentation of experimental procedures, samples collected, and analysis performed in this longitudinal dietary trial; Figure S2. Photos of the liver biopsy procedure; Figure S3. Morphometric measurements of Göttingen Minipigs fed ad libitum a high-risk (HR, *n* = 15) and a lower-risk (LR, *n* = 15) diet; Figure S4. Spearman correlations between the body weight and albumin levels in the plasma of Göttingen minipigs fed a high-risk (left panel) and a low-risk (right panel) diet.
