# Supplementary material for: Obesity Development and Signs of Metabolic Abnormalities in Young Göttingen Minipigs Consuming Energy Dense Diets Varying in Carbohydrate Quality
Source: Nutrients. 2021 May 6;13(5):1560. doi: 10.3390/nu13051560 (PMC8148203; doi:10.3390/nu13051560)
Supplement: Supplementary file 1 [file nutrients-13-01560-s001.zip › nutrients-1190860-supplementary.pdf]

nutrients

MDPI

Supplementary materials

Obesity development and signs of metabolic abnormalities in young Göttingen Minipigs consuming energy dense diets varying in carbohydrate quality

Mihai Victor Curtasu[1]*, Mette Skou Hedemann[1], Helle Nygaard Lærke[1] and Knud Erik Bach Knudsen[1]

[1] Department of Animal Science, Aarhus University, Blichers Alle 20, DK-8830 Tjele, Denmark;
* Correspondence: mihai.curtasu@anis.au.dk; Tel.: +4587154259

Table S1. Feed ingredients

	Diet	
	LR	HR
**Ingredients, g/kg (as-fed basis)***		
**Wheat starch**	232.8	232.8
**Whole grain wheat (milled)**	150	150
**Wheat bran (finely milled)**	125	125
**Wheat gluten**	65	65
**LT Fishmeal**	20	20
**HiMaize® 260 (60% resistant starch)**	200	-
**Fructose crystalline (food grade)**	-	200
**Lard 92/15**	150	150
**Monocalcium phosphate 17/22.7**	36.7	36.7
**Limestone (39 % Ca)**	9.0	9.0
**Salt (NaCl)**	6.5	6.5
**Lysine monohydrochloride**	1.7	1.7
**Choline chloride (70 %)**	1.3	1.3
**Vitamin premix ****	2.0	2.0

LR, low-risk diet; HR, high-risk diet
* Formulated to supply 55% energy from carbohydrates, 10% energy from protein and 35% energy from fat
** amount per kg additive: 1000 IE vitamin A, 1000 IE vitamin D3, 31500 mg alpha-tocopherol, 34615,4 mg DL-alphatocopherolacetate, 1050 mg vitamin B1, 1050 mg vitamin B2, 1575 mg vitamin B6, 10,5 mg vitamin B12, 5250 mg Ca-D-pantothenic acid, 10500 mg niacin, 26.25 mg biotin, 1050 mg vitamin K3, 42000 mg Fe as $FeSO_4$, 7500 mg Cu as $CuSO_4$, 21000 mg Mn as MnO.

Table S2. RT-PCR gene expression assays

Gene Symbol	Gene name	Assay ID1
	Carbohydrate metabolism	
SLC2A5	Solute Carrier Family 2 (Facilitated Glucose/Fructose Transporter) Member 5, Glut5	Ss03377332_u1
SLC2A4	Solute Carrier Family 2 (Facilitated Glucose Transporter) Member 4, Glut4	Ss03373325_g1
SLC2A8	Solute Carrier Family 2 (Facilitated Glucose Transporter) Member 8, Glut8	Ss03374161_m1
HK1	Hexokinase 1	Ss04323453_gH
FBP1	Fructose-Biphosphatase 1	Ss03393179_u1
PFKM	Phosphofructokinase, Muscle	Ss03380370_u1
	Fatty acid metabolism/ De Novo Lipogenesis	
ACACA	Acetyl-Coenzyme A Carboxylase Alpha	Ss03389962_m1
ACYL	ATP-citrate lyase	Ss03389566_m1
FASN	Fatty Acid Synthase	Ss03386194_u1
ADIPOR1	Adiponectin receptor 1	Ss03378803_u1
LEPR	Leptin Receptor	Ss03379257_u1
LEP	Leptin	Ss03392404_m1
ADIPOQ	Adiponectin	Ss03384375_u1
CIDEC	Cell Death-Inducing DFFA Like Effector C	Ss03389757_m1
	Lipid Transcription Factors	
PPARG	Peroxisome proliferator-activated receptor gamma	Ss03394829_m1
	Immune System/Inflammation	
CRP	C-reactive protein, pentraxin-related	Ss03390889_m1
IL6	Interleukin 6	Ss03384604_u1
TNF	Tumour Necrosis Factor	Ss03391318_g1
CCL5	C-C Motif Chemokine Ligand 5/ encodes RANTES	Ss03648939_m1
	Housekeeping genes	
GAPDH	Glyceraldehyde 3-phosphate dehydrogenase	Ss03375629_u1
HPRT1	Hypoxanthine phosphoribosyltransferase 1	Ss03388274_m1
ACTB	β-actin	Ss03376563_uH

[1] TaqMan Gene Expression Assay

Table S3. Plasma concentrations (ng/mL) of inflammation biomarkers after overnight fasting

					**P-value**[2]		
	Week	LR[1]	HR[1]	SEM	Diet	Time	Diet × Time
**IFNg**	4	4.69	4.46	0.64	NS	NS	NS
	12	4.37	3.97				
	20	4.6	4.61				
**IL2**	4	0.19	0.18	0.04	NS	NS	NS
	12	0.25	0.23				
	20	0.23	0.25				
**IL4**	4	0.35	0.49	0.14	NS	NS	NS
	12	0.51	0.76				
	20	0.65	0.41				
**IL10**	4	0.35	0.33	0.06	NS	NS	NS
	12	0.35	0.34				
	20	0.36	0.34				
**IL12**	4	0.79	0.78	0.06	NS	0.0003	NS
	12	0.79	0.9				
	20	0.63	0.65				
**IL18**	4	0.76	0.72	0.09	NS	NS	NS
	12	0.73	0.76				
	20	0.77	0.65				

[1]data presented as LS means; LR (n=15), HR (n=15)
[2]non-significant (NS), P > 0.1

Detailed RT-PCR protocol:

RNA purity and concentrations were measured by absorbance at 260-280 nm using a NanoDrop ND-8000 UV-vis spectrophotometer (NanoDrop Technologies, Wilmington, DE, USA). Superscript IV reverse transcriptase (Invitrogen, Taastrup, Denmark) together with oligo-dT and random primers were used to reverse-transcribe the purified RNA. An Esco Swift MaxPro Thermocycler (Holm & Halby, Brondby, Denmark) was used for cDNA synthesis by activation at 70°C for 10 min, followed by a run of 60 min at 42°C and followed by inactivation of the reaction at 70°C for 15 min. Liver, muscle, and AT cDNA samples were diluted 1:10, and standard samples were diluted 1:5. The standards were analyzed in triplicates using a dilution series of 1:4, whereas tissue samples were analyzed in duplicates. Tissue-specific genes were analyzed using TaqManTM Gene Expression Assays with pre-designed unlabelled PCR primers, TaqMan MGB probes labeled with fluorescein amidite (FAM) and TaqMan FAST Advanced Master Mix (Applied Biosystems, Stockholm, Sweden) on 384-well plates. A reaction volume of 10 μL was used, and RT-PCR protocol was as follows: 40 reaction cycles of amplification initiated at 95°C for 1 second, followed by a decrease to 60°C within 20 seconds of 1.6°C/second.

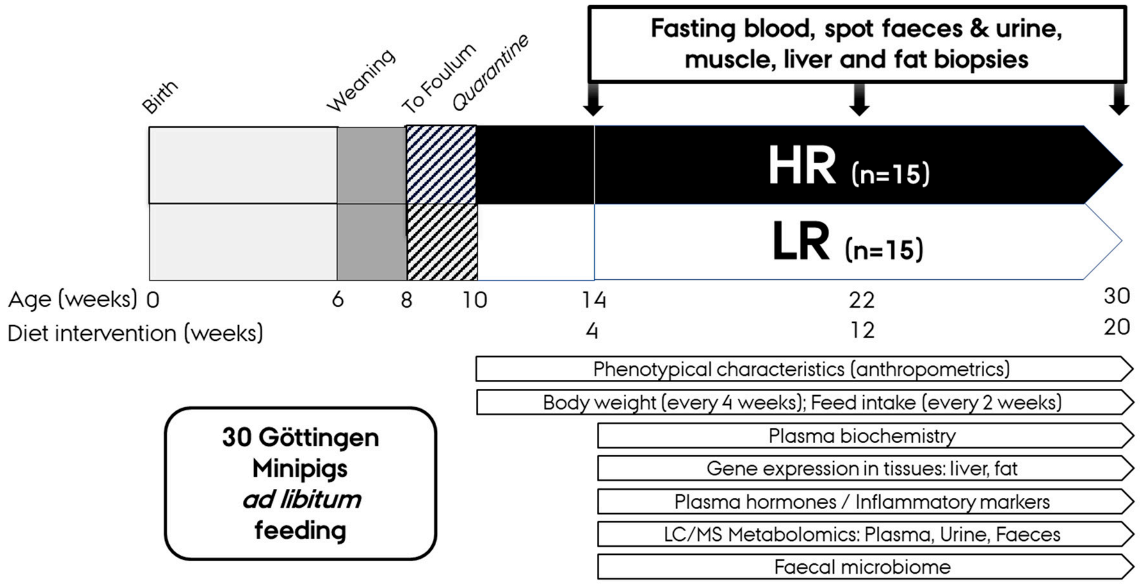

Figure S1. Flow chart presentation of experimental procedures, samples collected, and analysis performed in this longitudinal dietary trial. Note: LC/MS metabolomics and fecal microbiome analyses are presented in a separate publication.

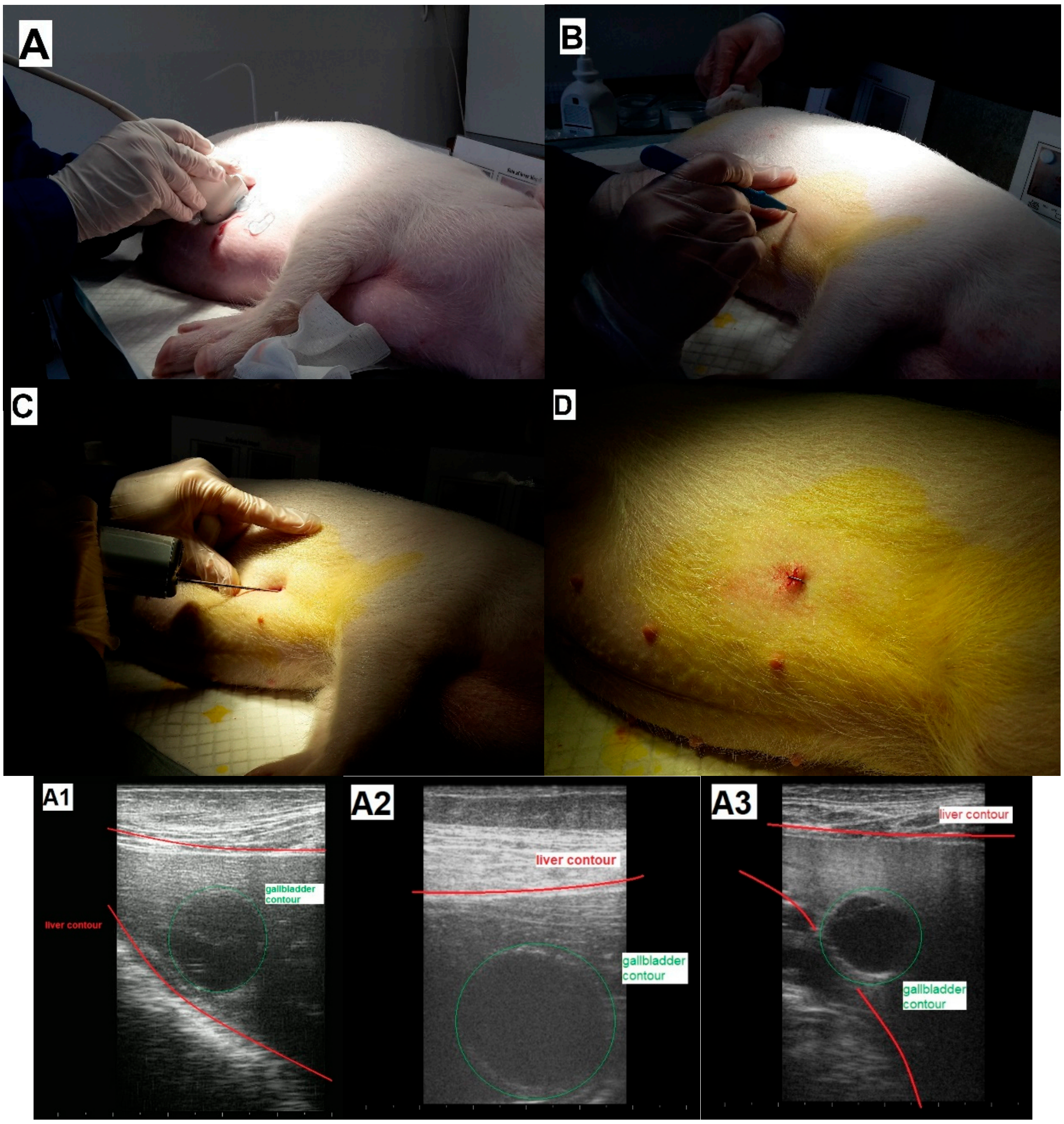

Figure S2. Photos of the liver biopsy procedure. A – ultrasound scanning of the liver to determine the position and volume of the gallbladder to avoid puncture during biopsy. Hair was removed prior to the procedure and after the ultrasound scanning the area was cleaned again and sterilized using A1-A3 - ultrasound view of the gallbladder (green) and liver delimitations (red line) in three different animals. B – Incision tipically made above the first tit of the minipig (for small/young minipigs) or between the first and the second tit (for larger animals; position differed based on the size of the animal and on the position of the gallbladder).. C – biopsy gun and biopsy sample collection. D – closure of the incision with surgical staples followed by an antiseptic wound spray.

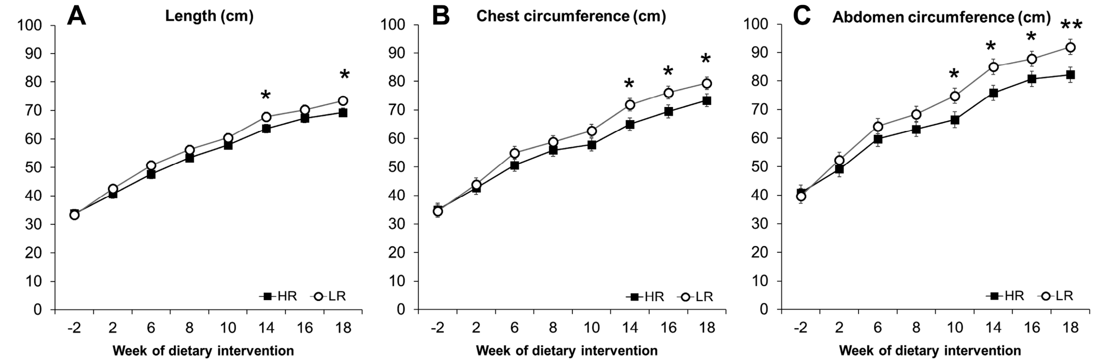

Figure S3. Morphometric measurements of Göttingen Minipigs fed ad libitum a high-risk (HR, n=15) and a lower-risk (LR, n=15) diet. (A) Measurements of length. (B) Chest circumference. (C) Abdomen circumference. Results are expressed as LS means, error bars indicating SEM. Significant difference (*P<0.05;**P<0.01).

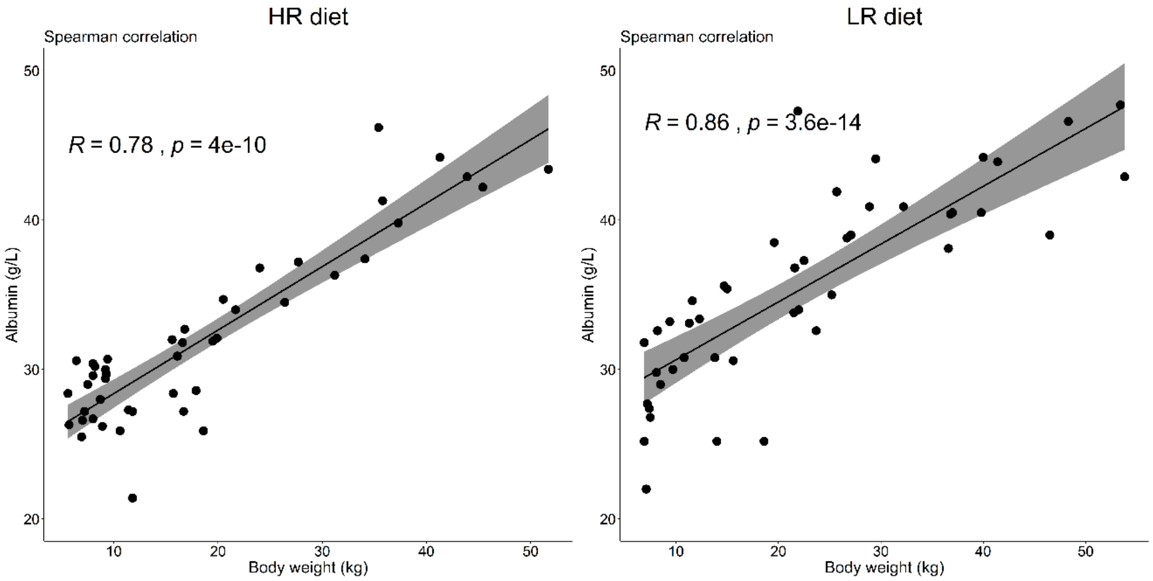

Figure S4. Spearman correlations between the body weight and albumin levels in the plasma of Göttingen minipigs fed a high-risk (left panel) and a low-risk (right panel) diet.
